# Comparative Genomic Analysis of *Lactobacillus plantarum*: An Overview

**DOI:** 10.1155/2019/4973214

**Published:** 2019-04-10

**Authors:** Eliane Evanovich, Patricia Jeanne de Souza Mendonça Mattos, João Farias Guerreiro

**Affiliations:** Laboratório de Genética Humana e Médica, Instituto de Ciências Biológicas-Universidade Federal do Pará, Pará, Brazil

## Abstract

**Background:**

*Lactobacillus plantarum* is widely used in the manufacture of dairy products, fermented foods, and bacteriocins. The genomes of the strains contain multiple genes which may have been acquired by horizontal gene transfer. Many of these genes are important for the regulation, metabolism, and transport of various sugars; however, other genes may carry and spread virulence and antibiotic resistance determinants. In this way, monitoring these genomes is essential to the manufacture of food. In this study, we aim to provide an overview of the genomic properties of *L. plantarum* based on approaches of comparative genomics.

**Results:**

The finding of the current study indicates that the core genome of *L. plantarum* presents 1425 protein-coding genes and is mostly related to the metabolic process. The accessory genome has on average 1320 genes that encodes protein involved in processes as the formation of bacteriocins, degradation of halogen, arsenic detoxification, and nisin resistance. Most of the strains show an ancestral synteny, similar to the one described in the genomes of *L. pentosus* KCA1 and *L. plantarum* WCFS1. The lifestyle island analyses did not show a pattern of arrangement or gene content according to habitat.

**Conclusions:**

Our results suggest that there is a high rate of transfer of genetic material between the strains. We did not identify any virulence factors and antibiotic resistance genes on the genomes. Thus, the strains may be useful for the biotechnology, bioremediation, and production of bacteriocins. The potential applications are, however, restricted to particular strains.

## 1. Background

*Lactobacillus plantarum* is a facultative heterofermentative lactic acid bacteria (LAB). It may obtain energy from different sugars, and it occupies an adaptive nutrient-rich niche such as gastrointestinal, vaginal, and urogenital tracts, vegetables, dairy products, and fermented foods [1–6]. This bacteria also carries relevant properties for not only the manufacture of a variety of food and wine but also vitamin production, bacteriocin, probiotic, antifungal, and potential anticaries agents [7–11]. In general, LAB has reduced genomes, but *L. plantarum* presents a larger genome with numerous genes, which have been acquired by horizontal gene transfer (HGT) mainly via mobile elements (prophages, plasmids, transposons, and integrons) [12, 13].

*L. plantarum* strains are capable of producing different antimicrobial compounds, such as hydrogen peroxide, organic acids (primarily lactic and acetic acid), antiaflatoxigenic, and bacteriocins [14–16]. The latter act against a wide range of bacterial pathogens, in the broad and narrow spectra [17, 18]. The plantaricins (or two-peptide bacteriocins) are usually produced by *L. plantarum* and include the plantaricins J51, JK, and EF [19–21].

Kleerebezem et al. [12] and Molenaar et al. [22] described in the *L. plantarum* WCFS1 genome a region known as the lifestyle island. It may have been acquired by HGT and is divided into two subregions of approximately 150 kb and 190 kb and contains several genes critical for the regulation, metabolism, and transport of sugars.

Mobile genetic elements are segments of DNA that can move within and between bacteria. They are potential disseminators of virulence factors and determinants of antibiotic resistance (AR). Several of these elements are found in the genomes of *L. plantarum* [23–27]; therefore, it is essential to screen these unwanted genes in the new strains. Previous studies have reported that AR genes have already been described in *L. crispatus*, *L. gasseri*, *L. reuteri*, and *L. plantarum* although their strains are considered safe in the United States through the Generally Recognized as Safe (GRAS) designation [28–35].

The aim of the paper is to provide an overview of the structural and genomic properties of *L. plantarum* genome strains available in the GenBank sequence database. We used complete genomes for avoiding the underestimation of gene content [36, 37]. The results show that the great majority of the mobile elements are Sha1 and Phig1 bacteriophages, originally isolated from *L. plantarum* [38, 39]. It suggests a high gene transfer rate between the strains. The outcomes also suggest a great potentiality in producing bacteriocins, except for the strains 16 and Zhang-LL. Therefore, the various applications of strains are unequivocal. In addition to their recognized applications, the strains may be useful to the pharmaceutical industry, in the bioremediation of halogenated pollutants and arsenic-rich soils.

## 2. Methods

### 2.1. Complete Genomes

The genome data was available in the NCBI (National Center for Biotechnology Information) (https://www.ncbi.nlm.nih.gov). Details regarding the identification and source of the samples used are in the additional file Table S1. Prokka version 1.12-beta [40] with the arguments kingdom Bacteria, and genus *Lactobacillus* was used for verifying the genome annotations.

### 2.2. Prediction of CRISPR Sequences and Mobile Elements

CRISPR sequences, mobile elements, genomic islands, and resistance genes were obtained via CRISPRFinder (http://crispr.i2bc.paris-saclay.fr/Server/) and PHAge Search Tool Enhanced Release (PHASTER) (http://phaster.ca) [40, 41] under the references intact score > 90, questionable score 70-90, and incomplete score < 70.

CARD (Comprehensive Antibiotic Resistance Database) (http://arpcard.mcmaster.ca/) was used to predict the resistome under the BLAST (Basic Local Alignment Search Tool), and RGI (Resistance Gene Identifier) (under Perfect hit, rigorous hit alone, and Perfect, Strict, and Loose hit criteria) [42]. The ResFinder 3.0 server (https://cge.cbs.dtu.dk/services/ResFinder/) was used to identify acquired antimicrobial resistance genes and/or chromosomal mutations [43].

### 2.3. Pan-genome

BPGA (Bacterial Pan Genome Analysis) tool [44] version 1.3 was used to identify core, accessory, and unique protein families. It was also used to search for the presence or absence of genes, phylogenetic inference, and atypical GC content and for mapping gene functions based on COG (Clusters of Orthologous Groups of proteins). The orthologous clusters were generated via USEARCH 9.2.64 (identity cut off = 50%) [45]. MUSCLE generated the alignments and the phylogenies [46], and gnuplot 4.6.6 (https://sourceforge.net/projects/gnuplot/files/latest/download source code freely distributed) was applied to plot the graphs [47].

### 2.4. Multiple Genome Alignment

Mauve, under progressiveMauve, was used to perform the synteny analyses and the multiple genome alignments (default setting) [48, 49]. *L. pentosus* KCA1 was used as the outgroup. Another analysis using the last subregion of the lifestyle island was also performed. In this, the value of minimal LCBs was equal to 1000.

## 3. Results and Discussion

### 3.1. Mobilome and Resistome

**Figure 1:**
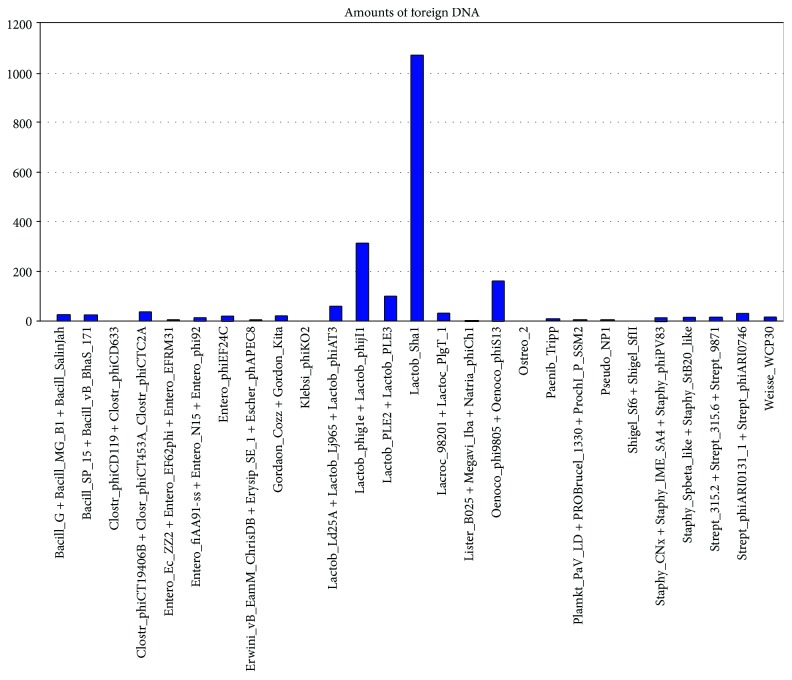
Types of prophage detected in *L. plantarum* genomes.

The results obtained via PHASTER showed that the sequences of bacteriophage origin have about 151 kb, i.e., about 48% of the size of the *L. plantarum* genomes. Bacteriophage proteins (DNA packaging protein, holin protein, lysin, tail, capsid, protease, terminase, and integrase) and hypothetical proteins were the most frequent (about 91%). The bacteriophages most encountered were Sha1 and Phig1, both isolated from *L. plantarum* [34, 35] as shown in Figure 1 and the additional file Table S2.

Nine of the 49 genomes display the CRISPR-Cas system (class 2, type II with four genes, cas9, cas1, cas2, and cns2, as found in *Streptococcus thermophilus*) [50]. These strains are from fermented foods (LY-78, MF1298, ZS2058, and TS12), raw milk (LZ206 and LZ227), an environmental sample (CLP0611), faeces of a newborn (ZJ316), and a cell culture (CGMCC 1.557). Length of the CRISPR sequence varies from 300 to 2111 bp, and the number of CRISPR spacers was four to 31. The degenerate repeat DR-consensus (5′-GTCTTGAATAGTAGTCATATCAAACAGGTTTAGAAC-3′) was equally reported in the *L. pentosus* MP-10 and *L. pentosus* KCA1 genomes [51, 52]. Evaluation of the spacer sequences revealed several invasion events by *Lactobacillus* bacteriophages (from *L. alimentarius* DSM 20249, *L. brevis* 925A, and *L. helveticus* FAM8627) and mainly by *L. plantarum* bacteriophages. This result is consistent with what was obtained via PHASTER and suggests that the CRISPR-Cas system is not the primary defence against bacteriophage invasion (additional file: Table S3). According to Abriouel et al. [51], the presence of bacteriophages may provide some selective advantage to the bacterial cell, by helping in the fight against other prophage infections. The domestication of of mobile genetic elements, which is useful for different bacterial processes, has been described [53–56], and it may also be applied to *Lactobacillus*, including *L. plantarum*.

ResFinder, CARD, and PATRIC did not indicate potential antibiotic resistance or virulence determinants. Most *L. plantarum* strains have putative genes annotated as antibiotic resistance genes or a virulence factor (such as the putative formate acetyltransferase 3-ybiW gene or mdxE-maltodextrin ABC-transporter protein gene), but they seem to be only spurious partial hits [40], which may exert other cellular functions. AR genes were not detected in the plasmids, as well.

### 3.2. Pan-genome

**Figure 2:**
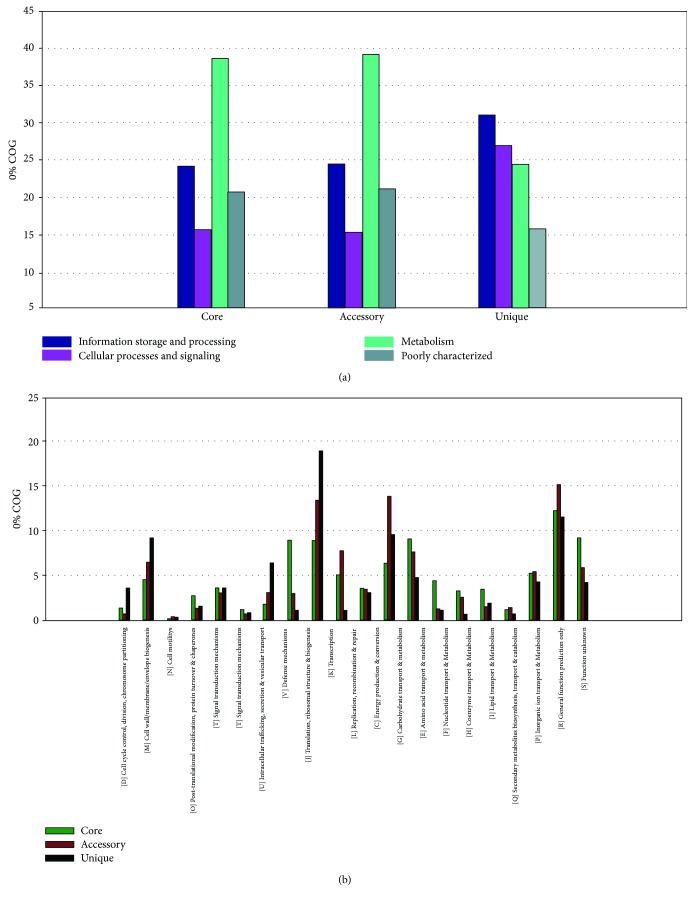
Pan-genome prediction. (a) core, accessory and unique genes into the functional standard of the COG; (b) the specific distribution of the genes into 20 COG categories.

On average, the genomes present 2917 protein-coding genes, 1425 of which belong to the core genome. Most core orthologous groups (OGs) are related to metabolism. OG distribution in COG categories is shown in Figure 2. This result is not surprising, due to the lifestyle island [3, 4, 57]. Other important OGs from the core genome are protein-coding genes involved in the synthesis of exopolysaccharides (EPS), histidine protein kinase (HPK), L-2-haloacid dehalogenase, sortase A (srtA), and fibrinogen-binding. They are essential genes to the synthesis of plantaricin and degradation of halogenated compounds and for host-bacterial interaction [58–64].

Hpk6, hpk7, and hpk11 proteins, belonging to histidine protein kinase (HPK), have a regulatory function in the synthesis of plantaricins and are therefore crucial to the strains [61]. The *deh* gene encodes an L-2-haloacid dehalogenase, an enzyme that degrades halogenated compounds present in drugs and environmental pollutants such as chlorobenzene, chlorocyclohexane, chloroalkane, and chloroalkene [62]. This enzyme presents applications in chemical industries, bioremediation, and sustainable chemistry [60, 61]. Results obtained for the pan-genome are shown in the additional file Table S4.

Genome analysis indicates that an efficient system for arsenic detoxification is restricted to *L. plantarum* WCFS1. This mechanism is regulated by the *arsR* gene and depends on ArsD, ArsA, and ArsB proteins [12, 65]. The other strains contain only *arsC* and *arsR* genes, and therefore, they have the arsenic partial detoxification [64].

The nisin (nsr) gene is found in all strains analyzed in this study but encodes a protein truncated in ten strains (16, 5-2, JDM1, MF1298, p-8, ST-III, TMW 1.25, TMW 1.277, WCFS1, and ZJ316). In a similar way, Sun et al. [66] described in *L. lactis* a truncated nisin protein, with the activity reduced. Hence, it is expected that these strains also show a reduced nisin activity.

The production of vitamins in the food industry is greatly exploited by food biotechnology. However, *L. plantarum* is deficient in the production of vitamin B complex biotin (B7), niacin (B3), pantothenate (B5), and pyridoxine (B6) [67, 68]. But it produces large amounts of folate (B9) [69, 70]. Folate gene clusters are described by Kleerebezem et al. [12] in the *L. plantarum* WCFS1 genome. This cluster presents nine genes (*folA*, *folB*, *folC1*, *folC2*, *folD*, *folE*, *folK*, *folP*, and *folQ*) identified in the core genome of all strains analyzed here. In contrast, the presence of the ribo genes (*ribA*, *ribB*, *ribD*, and *ribH*) required for riboflavin synthesis is restricted to ten strains (the ATCC 8014, JDM1, KC28, LPL-1, TMW 1.25, TMW 1.277, TMW 1.708, TMW 1.1623, X7021, and ST-III strains) originally isolated from food. Other strains present a rib operon incomplete and probably do not produce riboflavin [71].

Besides the production of nisin and vitamins, the monitoring of the production of biogenic amines (BA) by LAB is also of paramount importance to the food industry. BA, such as putrescine and spermidine, are nitrogen compounds formed during the decarboxylation of amino acids by bacteria [72]. They are toxic when accumulated in food processing and storage, causing human health problems [73, 74]. In this way, the ability to produce large amounts of BA may be an obstacle to the use of some LAB. Alan et al. [75] monitored the ability of *L. plantarum* JDM1 to produce metabolites based on the decarboxylase test and its genic content. The authors concluded that the presence of the glutamate decarboxylase (*gadB*) gene is not enough to produce BA.

In this work, we observed that the gene encoding in the enzyme glutamate decarboxylase is common to all the strains analyzed. Another decarboxylase gene, *panD* (encoded 1-decarboxylase aspartate), was also found in the *L. plantarum* B21, *L. plantarum* TMW 1.708, and *L. plantarum* WCFS1 genomes. Based on genomic analysis, it is not possible to assess whether the amounts of BA produced are deleterious, but analyses in culture medium show that they are not [75, 76].

Only one plantaricin (*pln*) gene was identified in the core genome. This gene encodes a bacteriocin immunity protein with 88 amino acids (lp_2952 in reference genome WCFS1). Other genes are restricted to the accessory genome and unique gene families. The accessory genome has an average of 1320 OGs, mostly related to the phosphotransferase system (PTS) and biosynthesis of amino acids. PTS proteins transport substance into the cell, including carbohydrates. The sugar-specific transport of these proteins explains their greater genetic representation within the accessory genome of the *L. plantarum* strains.

A protein-coding gene involved in the export of bacteriocins, the bacteriocin ABC-transporter gene was found in most samples (except in the Asian strains GB-LP1, JBE490, LPL-1, LZ206, LZ227, Zhang-LL, and ZJ316). The mannose-specific adhesin (*msa*) gene also belongs to the accessory genome, being present in 22 strains extracted from different sources (fermented foods, flies, saliva, cell culture, faeces, environment, and probiotics). In addition, the collagen binding protein (*cnaB*) gene encodes an adhesion, likely related to colonisation and competition against pathogenic bacteria, an important feature of probiotic strains [77–79].

**Figure 3:**
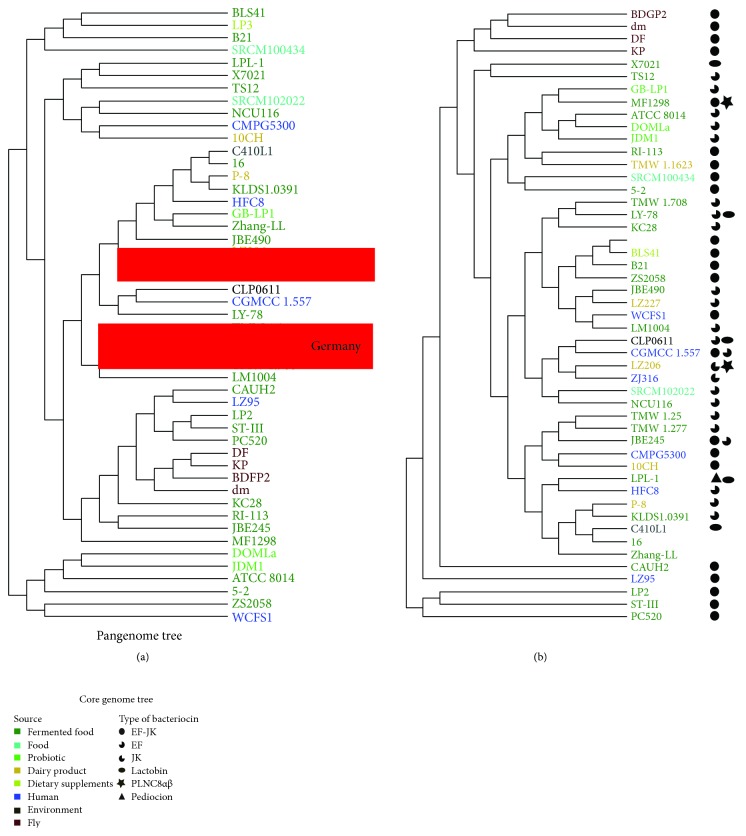
Phylogenies based on pangenome analyses of *L. plantarum*. (a) A phylogenetic tree constructed by pangenome data. The branches highlighted indicate the geographical locations where the strains were isolated. (b) A phylogenetic tree based on core genome data. The first box shows which colors correspond to the source of the strains, and the box below described the symbols used here to represent the bacteriocins. The scales below the trees correspond to the time in millions of years (Mya). Nisin bacteriocin is not shown since it is present in most strains.

Phylogenetic trees obtained from the pan-genome and core genome are shown in Figures 3(a) and 3(b), respectively. The core genome tree recovered better the phylogenetic relationships between the strains (reference NCBI-Genome Tree Report: https://www.ncbi.nlm.nih.gov/genome/tree/1108?). In contrast, the pan-genome tree may show genomic novelties, such as the gaining of new genes by HGT [80, 81]. The monophyly of the strains extracted from flies and from potential probiotics was recovered in the phylogenetic trees. Two branches highlighted in Figure 3(a) indicate strains grouped according to the geographical location where they were isolated. *L. plantarum* LZ206, ZJ316, and LZ227 strains are from Hangzhou, China, and have the same GC content (45.2%). The milk strains LZ206 and LZ227 share a CRISPR spacer (AAACGTTCTATGCTTCGTTTCCTCAGCATC) and are also the final part of a 74.2 kb foreign fragment. This may suggest a shift of genetic material between them. The origin of the cluster formed by *L. plantarum* TMW 1.25+TMW 1.277 (monophyletic group), TMW 1.1623, and TMW 1.708 strains from Germany appears to be more complex than those of the other group. It has a similar CG content (between 45.2 and 45.4%) but does not have CRISPR sequences that could indicate recent invasions. Based on bacteriophage analysis, we were able to identify that the *L. plantarum* TMW 1.1623 strain partially shares with *L. plantarum* TMW 1.708 a 44.2 kb fragment (on positions 1129520 to 1173731), mainly containing the bacteriophage Lactob_Sha1 (35) and Lactob_JCL1032 (8), while with the *L. plantarum* TMW 1.25 and TMW 1.277 strains, it shares in a region of about 47.3 kb (positions 2074572-2121906), composed mainly of bacteriophages of the types Oenoco_phiS13 (16), Oenoco_phi9805 (15), and Lactob_Lj965 (14).

### 3.3. Bacteriocin Genes

Twenty-one strains present EF and JK plantaricin genes, which make up the pln cluster (formed by 25 genes). However, *L. plantarum* 16, *L. plantarum* C410L1, and *L. plantarum* subsp. *plantarum* p-8 strains have a frameshift in the *plnE* gene, and thus, the synthesis of EF plantaricin by them is questionable. These strains contain IS3 and IS256 insertion elements (IS) in the middle of the pln cluster, suggesting that IS may be related to the loss of function in the *plnE* gene. The bacteriocin genes present in the strains are shown in Figure 3(b).

Capy et al. [82], Schneider and Lenski [83], and Eraclio et al. [84] proposed that IS could have an adaptive function and play a significant role in the chromosomal rearrangement. This assertion is likely persuasive since IS and transposable elements can inactivate, insert, delete, or displace operons and gene cassettes, shifting the adaptive value of the microorganism within its habitat.

Protein-coding genes for class IIb bacteriocin, a lactobin A/cerein 7B protein, are restricted to CLP0611, JBE245, LPL-1, LY-78, Z227, and CGMCC 1.557 strains. PLNC8*αβ* genes expressed in *L. plantarum* NC8 [85] were also found in the genomes of MF1298 (fermented sausage, Norway) and LZ206 (cow milk, China) strains. PLNC8*αβ* proved effective in controlling *Porphyromonas gingivalis*, a bacterium that causes periodontitis [85, 86]. Thus, products based on the LZ206 and MF1298 strains may be potentially useful for the treatment of periodontitis. LPL-1 presents pediocin PA-1 bacteriocins (class IIa), similar to that found in "*Pediococcus acidilactici H*".

The origin of some bacteriocins is attributed to defective bacteriophage proteins, such as the R-type pyocin related to the P2 bacteriophage, carotovoricin to tail-like bacteriocin, and monocins to TP901-1-like bacteriophage tails [87–90]. We found no evidence that bacteriocins are bacteriophage-derived proteins; however, these proteins may be important in rearrangement and environmental adaptation [72–74].

### 3.4. Multiple Genome Alignments

**Figure 4:**
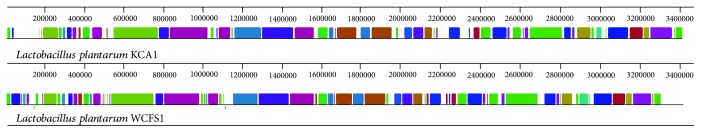
Genome comparison between *L. plantarum* WCFS1 and *L. pentosus* KCA1. The colored blocks correspond to LCBs.

**Figure 5:**
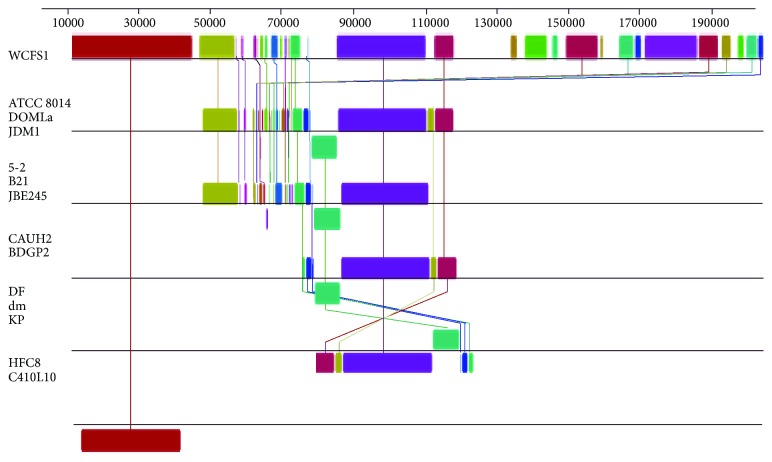
Comparative patterns of the lifestyle island in *L. plantarum* strains. The boxes below or above on the LCBs mean that the gene clusters are associated with obtaining energy. LCB weight value was 1000 bp. The region was analyzed corresponding to lp_3131 to lp_3661 of the *L. plantarum* WSFS1 genome.

The genomic arrangement possesses small variations among strains, mainly in the lifestyle island (Figure 4). We also conducted a comparative analysis using the lifestyle island (lp_3131 to lp_3661 position genes, using *L. plantarum* WCFS1 as reference) [3, 4, 23, 91]. The analysis of this genomic region did not show a pattern associated with the habitat of the strains (Figure 5). Some arrangements were consistent with the phylogenetic relationship shown in Figure 3(b), for instance, *L. plantarum* ATCC 8014, DOMLa, and JDM1 strains, while other similar arrangements arose via HGT.

## 4. Conclusions

*L. plantarum* strains are potentially useful in biotechnology, bioremediation, and pharmaceutical products and in the manufacture of bacteriocins. None of the strains has antibiotic resistance genes or virulence factors. But the genomic screening of new strains is essential because the bacterial genomes are dynamic entities. HGT seems to play a large role on genomic innovations, and it may be related to the great adaptability of the *L. plantarum* to different ecological niches. In contrast, we found no evidence on the adaptive role of the lifestyle island.

## Data Availability

The supporting data are enclosed as additional files.

## Abbreviations

*arsC* gene: Arsenate reductase gene
*arsR* gene: Arsenical resistance operon transcriptional repressor gene
BA: Biogenic amines
*cnaB* gene: Collagen binding protein gene
*deh* gene: L-2-haloacid dehalogenase gene
HGT: Horizontal gene transfer
HPK: Histidine protein kinase
LCBs: Locally collinear blocks
*msa* gene: Mannose-specific adhesin gene
*nsr* gene: Nisin gene
*pln* gene: Plantaricin gene
*srtA* gene: Sortase A gene.

## Additional Points

*Authors' Information*. This manuscript is part of the Ph.D. thesis of Eliane Evanovich from Programa de Pós-Graduação em Genética e Biologia Molecular, Universidade Federal do Pará.

## Supplementary Materials

Supplementary 1 Table S1: the table shows the description of the samples used in the manuscript.

Supplementary 2 Table S2: the characteristics of the phages found in the genomes.

Supplementary 3 CRISPR sequences present in the analyzed genomes.

Supplementary 4 Detailed information on the pan-genome analysis.
