# Supplementary material for: Comparative Genomic Analysis of Lactobacillus plantarum: An Overview
Source: Int J Genomics. 2019 Apr 10;2019:4973214. doi: 10.1155/2019/4973214 (PMC6481158; doi:10.1155/2019/4973214)
Supplement: Supplementary 1 — Table S1: the table shows the description of the samples used in the manuscript. [file 4973214.f1.pdf]

Table S1. Characteristics of the *50* Lactobacillus

used in this study.

**Organism name**	**Isolation source**	**Access Number**	**References**
Lactobacillus pentosus KCA1	Vagina– Nigeria	NZ_CM001538.1	1
L. plantarum 10CH	Cheese- United Kingdom	CP023728.1	2
L. plantarum 16	Isolated from malt production– Ireland	NC_021514.1	3
L. plantarum 5-2	Fermented soybean- China	NZ_CP009236.1	4
L. plantarum ATCC 8014	Green olive fermentation- Spain	NZ_CP024413.1	Unpublished
L. plantarum B21	Vietnamese Fermented Sausage- Vietnam	NZ_CP010528.1	5
L. plantarum BDGP2	*Drosophila melanogaster*- United States	NZ_CP023174.1	6
L. plantarum C410L1	Pit mud- China	NZ_CP017954.1	Unpublished
L. plantarum CAUH2	Sichuan pickle vegetables– China	NZ_CP015126.1	7
L. plantarum CLP0611	Environment- South Korea	NZ_CP019722.1	Unpublished
L. plantarum CMPG5300	Vagina of healthy woman- *Homo sapiens*– Belgium	NZ_CM002918.1	8
L. plantarum DF	*Drosophila melanogaster*- Canada	NZ_CP013753.1	9
L. plantarum dm	*Drosophila melanogaster*- United States	NZ_CP022373.1	10
L. plantarum DOMLa	Probiotic potential– China	NZ_CP004406.1	Unpublished
L. plantarum GB-LP1	Traditional Korean Fermented Food- South Korea	NZ_CP020564.1	11
L. plantarum HFC8	Faecal– *Homo sapiens*- India	NZ_CP012650.1	12
L. plantarum JBE245	Meju- South Korea	NZ_CP014780.1	13
L. plantarum JBE490	Nuruk - South Korea	NZ_CP020861.1	Unpublished
L. plantarum JDM1	Commercial LAB with several probiotic functions– China	NC_012984.1	14
L. plantarum KC28	Fermented Kimchi- South Korea	CP026743.1	Unpublished

L. plantarum KLDS1.0391	Nuruk – South Korea	CP019348.1	15
L. plantarum KP	*Drosophila melanogaster*– Canada	CP019348.1	9
L. plantarum LM1004	Napa cabbage kimchi- South Korea	CP025988.1	Unpublished
L. plantarum LP2	Pickles– China	NZ_CP020816.1	Unpublished
L. plantarum LP3	Commercial dietary supplements- South Korea	NZ_CP017066.1	Unpublished
L. plantarum LPL-1	Fermented fish– China	NZ_CP021997.1	16
L. plantarum LY-78	Fermented chinese cabbage– China	NZ_CP015308.1	Unpublished
L. plantarum LZ206	Cow milk– *Bos taurus*– China	NZ_CP015966.1	17
L. plantarum LZ227	Cow milk– *Bos taurus*– China	NZ_CP015857.1	18
L. plantarum LZ95	Faecal- *Homo sapiens*– China	NZ_CP012122.1	19
L. plantarum MF1298	Fermented sausage– Norway	NZ_CP013149.1	Unpublished
L. plantarum NCU116	Chinese pickle– China	NZ_CP016071.1	Unpublished
L. plantarum PC520	Chinese fermented food pickles- China	NZ_CP023772.1	Unpublished
L. plantarum RI-113	Fermented salami– Switzerland	NZ_CP017406.1	Unpublished
L. plantarum SRCM102022	Food- South Korea	NZ_CP021501.1	Unpublished
L. plantarum TMW 1.25	Raw sausage– Germany	NZ_CP017354.1	20
L. plantarum TMW 1.277	Palm wine– Germany	NZ_CP017363.1	20
L. plantarum TMW 1.708	Raw sausage– Germany	NZ_CP017374.1	20
L. plantarum TMW 1.1623	Cow Milk– *Bos taurus*– Germany	NZ_CP017379.1	20
L. plantarum WCFS1	Saliva– *Homo sapiens* – United States	NC_004567.2	21
L. plantarum X7021	Brine of stinky tofu- China	CP025412.1	Unpublished
L. plantarum Zhang-LL	Fermented rice- China	NZ_CP011769.1	Unpublished
L. plantarum ZJ316	Faecal- *Homo sapiens* – China	NC_020229.1	22
L. plantarum ZS2058	Sauerkraut – China	NZ_CP012343.1	23

L. plantarum subsp. plantarum BLS41	Kimchi- South Korea	NZ_CP018209.1	Unpublished
L. plantarum subsp. plantarum CGMCC 1.557	Intestine- *Homo sapiens*– China	NZ_CP016270.1	24
L. plantarum subsp. plantarum P-8	Traditional dairy products– China	NC_021224.2	25
L. plantarum subsp. plantarum SRCM100434	Food- South Korea	NZ_CP021528.1	Unpublished
L. plantarum subsp. plantarum ST-III	Kimchi- China	NC_014554.1	26
L. plantarum subsp. plantarum TS12	Stinky Tofu– Malaysia	NZ_CP018324.1	Unpublished

References

1. Anukam KC, Macklaim JM, Gloor GB, Reid G, Boekhorst J: **Genome sequence of *Lactobacillus pentosus* KCA1: vaginal isolate from a healthy premenopausal woman.** *PLoS ONE*. 2013, 8:E59239.

2. El Halfawy NM, El-Naggar MY, Andrews SC: **Complete genome sequence of *Lactobacillus plantarum* 10CH, a potential probiotic lactic acid bacterium with potent antimicrobial activity.** *Genome Announc* 2017, 5: e01398-17.

3. Crowley S, Bottacini F, Mahony J, Van Sinderen Douwe: **Complete Genome Sequence of *Lactobacillus plantarum* strain 16, a broad-spectrum antifungal-producing lactic acid bacterium.** *Genome Announc* 2013, 1: e00533-13.

4. Liu CJ, Wang R, Gong FM, Liu XF, Zheng HJ, Luo YY, Li XR: **Complete genome sequences and comparative genome analysis of *Lactobacillus plantarum* strain 5-2 isolated from fermented soybean.** *Genomics* 2015, 106:404-411.

5. Golneshin A, Adetutu E, Ball AS, May BK, Van TT, Smith AT: **Complete Genome Sequence of *Lactobacillus plantarum* strain B21, a bacteriocin-producing strain isolated from Vietnamese fermented sausage nem chua.** *Genome Announc* 2015, 3: pii: e00055-15.

6. Kenneth H, Wan CY, Park S, Hammonds AS, Booth BW, Celniker SE: **Complete genome sequence of *Lactobacillus plantarum* Oregon-R-modENCODE strain BDGP2 isolated from *Drosophila melanogaster* gut.** *Genome Announc* 2017, 5: e01155-17.

7. Yang Y, Yin J, Liu J, Xu Q, Lan T, Ren F, Hao Y: **The copper homeostasis transcription factor copR is involved in H2O2 stress in *Lactobacillus plantarum* CAUH2.** *Front Microbiol* 2017, 8:2015.

8. Malik S, Siezen RJ, Renckens B, Vaneechoutte M, Vanderleyden J, Lebeer S: **Draft genome sequence of *Lactobacillus plantarum* CMPG5300, a human vaginal isolate.** *Genome Announc* 2014, 2: e01149-14.

9. Petkau K, Fast D, Duggal A, Foley E: **Comparative evaluation of the genomes of three common *Drosophila*-associated bacteria.** *Biol Open* 2016, 5: 1305-1316.

10. Simhadri RK, Fast EM, Guo R, Schultz MJ, Vaisman N, Ortiz L, Bybee J, Slatko BE, Frydman HM: **The gut commensal microbiome of *Drosophila melanogaster* is modified by the endosymbiont Wolbachia.** *MSphere* 2017, 2: e00287-17.

11. Yu J, Ahn S, Kim K, Caetano-Anolles K, Lee C, Kang J, Cho K, Yoon SH, Kang DK, Kim H: **Comparative genomic analysis of *Lactobacillus plantarum* GB-LP1 isolated from traditional Korean fermented food.** *J Microbiol Biotechnol* 2017, 27:1419-1427.

12. Kumari M, Swarnkar MK, Kumar S, Singh AK, Gupta M: **Complete genome sequence of potential probiotic *Lactobacillus* sp. HFC8, isolated from human gut using PacBio SMRT sequencing.** *Genome Announc* 2015, 3:e01337-15.

13. Heo J, Uhm T: **Complete genome sequence of *Lactobacillus plantarum* JBE245 isolated from Meju.** *Kor. J. Microbiol* 2017, 53:344-346.

14. Zhang ZY, Liu C, Zhu YZ, Zhong Y, Zhu YQ, Zheng HJ, Zhao GP, Wang SY, Guo XK: **Complete genome sequence of *Lactobacillus plantarum* JDM1**. *J Bacteriol* 2009, 191:5020-1.

15. Jia FF, Zhang LJ, Pang XH, Gu XX, Abdelazez A, Liang Y, Sun SR, Meng XC: **Complete genome sequence of bacteriocin-producing *Lactobacillus plantarum* KLDS1.0391, a probiotic strain with gastrointestinal tract resistance and adhesion to the intestinal epithelial cells.** *Genomics* 2017, 109:432-437.

16. Wang Y, Shang N, Qin Y, Zhang Y, Zhang J, Li P: **The complete genome sequence of *Lactobacillus plantarum* LPL-1, a novel antibacterial probiotic producing class IIa bacteriocin.** *J Biotechnol* 2018, 266:84-88.

17. Li P, Gu Q, Zhou Q: **Complete genome sequence of *Lactobacillus plantarum* LZ206, a potential probiotic strain with antimicrobial activity against food-borne pathogenic microorganisms.** *J Biotechnol* 2016, 238:52-55.

18. Li P, Zhou Q, Gu Q: **Complete genome sequence of *Lactobacillus plantarum* LZ227, a potential probiotic strain producing B-group vitamins.** *J Biotechnol* 2016, 234:66-70.

19. Li P, Gu Q: **Complete genome sequence of *Lactobacillus plantarum* LZ95, a potential probiotic strain producing bacteriocins and B-group vitamin riboflavin.** *J Biotechnol* 2016, 229:1-2.

20. Kafka TA, Geissler AJ, Vogel RF: **Multiple genome sequences of *Lactobacillus plantarum* strains.** *Genome Announc* 2017, 5: e00654-17.

21. Siezen RJ, Francke C, Renckens B, Boekhorst J, Wels M, Kleerebezem M, Van Hijum SA: **Complete resequencing and reannotation of the *Lactobacillus plantarum* WCFS1 genome.** *J. Bacteriol* 2012, 194:195-196.

22. Li X, Gu Q, Lou X, Zhang X, Song D, Shen L, Zhao Y: **Complete genome sequence of the probiotic *Lactobacillus plantarum* strain ZJ316.** *Genome Announc* 2013, 1:E0009413.

23. Yang B, Chen H, Tian F, Zhao J, Gu Z, Zhang H, Chen YQ, Chen W: **Complete genome sequence of *Lactobacillus plantarum* ZS2058, a probiotic strain with high conjugated linoleic acid production ability.** *J Biotechnol* 2015, 214:212-213.

24. Ren D, Li C, Qin Y, Yin R, Du S, Ye F, Liu C, Liu H, Wang M, Li Y, Sun Y, Li X, Tian M, Jin N: **In vitro evaluation of the probiotic and functional potential of *Lactobacillus* strains isolated from fermented food and human intestine.** *Anaerobe* 2014, 30:1e10.

25. Zhang W, Sun Z, Bilige M, Zhang H: **Complete genome sequence of probiotic *Lactobacillus plantarum* P-8 with antibacterial activity.** *J Biotechnol* 2015, 193:41-42.

26. Wang Y, Chen C, Ai L, Zhou F, Zhou Z, Wang L, Zhang H, Chen W, Guo B: **Complete genome sequence of the probiotic *Lactobacillus plantarum* ST-III.** *J. Bacteriol* 2011, 193:313-314.
