# Supplementary material for: Comparative Genomic Analysis of Lactobacillus plantarum: An Overview
Source: Int J Genomics. 2019 Apr 10;2019:4973214. doi: 10.1155/2019/4973214 (PMC6481158; doi:10.1155/2019/4973214)
Supplement: Supplementary 2 — Table S2: the characteristics of the phages found in the genomes. [file 4973214.f2.pdf]

Table S2. Characteristics of the prophage regions identified by PHASTER. Plasmids are indicated in italic. Prophage regions with score bigger than 90 are Intact, with the score equal to 70-90 are questionable and score less than 70 are incomplete.

| Sample | Length (Kb) | Score | Total Proteins | Phage+Hypothetical proteins (%) | Region Position | Most Common Phage | GC % |
|---|---|---|---|---|---|---|---|
| 10CH | 41.9 | 150 | 54 | 100 | 1012923-105485 | Lactob_Sha1_NC_019489(25) | 40.4 |
| | 26 | 50 | 13 | 84.6 | 1854948-1880969 | Oenoco_phiS13_NC_023560(1) | 42.3 |
| | 50 | 100 | 61 | 93.4 | 2163582-2213652 | Lactob_phig1e_NC_004305(9) | 93.4 |
| 16 | 7.6 | 50 | 10 | 76.9 | 749653-757341 | Bacill_MG_B1(1) | 44.2 |
| | 83.9 | 150 | 91 | 76.9 | 2013949-2097849 | Lactob_Sha1(10) | 41.6 |
| *Lp16H* | 17.1 | 60 | 10 | 80.0 | 13447-30547 | Lactob_phiAT3(2) | 37.3 |
| 5-2 | 44.1 | 150 | 52 | 96.1 | 975405-1019555 | Lactob_Sha1_NC_019489(19) | 41.5 |
| | 42.3 | 90 | 57 | 92.9 | 1487964-1530300 | Lactob_phig1e_NC_004305(13) | 39.6 |
| | 45.6 | 150 | 57 | 96.4 | 2147045-2192663 | Lactob_phig1e_NC_004305(22) | 42.2 |
| ATCC 8014 | 16.9 | 100 | 23 | 95.0 | 37804-54802 | Entero_IME_EFm5_NC_028826 (4) | 42.4 |
| | 38.9 | 140 | 55 | 96.3 | 512764-551706 | Bacill_vB_BhaS_171_NC_030904(15) | 41.5 |
| | 42.7 | 150 | 52 | 98.0 | 1020394-1063100 | Lactob_Sha1_NC_019489(18) | 41.5 |
| | 16.1 | 20 | 7 | 98.0 | 1093207-1109313 | Entero_phi92_NC_023693(4) | 41.2 |
| *pLP39* | 38.9 | 140 | 49 | 95.9 | 48-38990 | Lactob_PLE2_NC_031036(15) | 41.5 |
| B21 | 44.6 | 150 | 47 | 100 | 1854269-1898886 | Lactob_Sha1_NC_019489(34) | 40.5 |
| | 38.1 | 110 | 57 | 100 | 2195065-2233217 | Lactob_Lj965_NC_005355(16) | 42.2 |
| BDGP2 | 52.8 | 110 | 61 | 90.1 | 180409-233281 | Lactob_PLE3_NC_031125(14) | 41.7 |
| | 47.3 | 100 | 50 | 90.0 | 240834-288168 | Oenoco_phiS13_NC_023560(16) | 41.2 |
| | 41.7 | 150 | 53 | 100 | 1182043-1223768 | Lactob_Sha1_NC_019489(25) | 41.8 |
| | 45.1 | 150 | 55 | 96.3 | 1411967-1457123 | Lactob_Sha1_NC_019489(35) | 41.6 |

| | | | | | | | |
|---|---|---|---|---|---|---|---|
| | 42.2 | 80 | 58 | 93.1 | 1534710-1576934 | Lactob_phig1e_NC_004305(11) | 42.3 |
| | 16.5 | 70 | 22 | 100 | 2663883-2680422 | Lactob_Sha1_NC_019489(3) | 41.1 |
| | 38.9 | 150 | 56 | 98.2 | 3203218-3242183 | Lactob_phig1e_NC_004305(22) | 42.3 |
| *pLtBDGP2A* | 27.6 | 40 | 10 | 60.0 | 14743-42405 | Klebsi_phiKO2_NC_005857(2) | 36.8 |
| *pLtBDGP2D* | 5.2 | 50 | 9 | 60.0 | 6413-11702 | Strept_315.6_NC_004589(2) | 36.6 |
| C410L1 | 61.7 | 150 | 74 | 97.2 | 383584-445349 | Lactob_phig1e_NC_004305(21) | 41.6 |
| | 50.8 | 150 | 56 | 100 | 1314057-1364912 | Lactob_Sha1_NC_019489(25) | 42.4 |
| *unnamed1* | 6 | 70 | 9 | 66.6 | 48793-54826 | Staphy_SPbeta_like_NC_029119(2) | 41.4 |
| *unnamed3* | 30.4 | 60 | 34 | 47.0 | 1-30457 | Strept_phiARI0131_2_NC_031941(5) | 45.8 |
| *unnamed6* | 26.6 | 30 | 32 | 62.5 | 1-26663 | Erwini_vB_EamM_ChrisDB_NC_031126(3) | 36.8 |
| CAUH2 | 41.4 | 130 | 57 | 94.7 | 555724-597202 | Lactob_phig1e_NC_004305(10) | 40.2 |
| | 23.7 | 50 | 13 | 84.6 | 821526-845245 | Lactob_PLE3_NC_031125(3) | 41.0 |
| | 32.4 | 100 | 32 | 100 | 2135406-2167806 | Oenoco_phiS13_NC_023560(16) | 42.8 |
| | 16.2 | 20 | 28 | 100 | 2170950-2187222 | Lactob_phig1e_NC_004305(6) | 40.6 |
| *pCAUH203* | 7.9 | 20 | 11 | 81.8 | 1-7995 | Bacill_SP_15_NC_031245(1) | 36.4 |
| CLP0611 | 54.9 | 150 | 48 | 100 | 1755604-1810548 | Lactob_Sha1_NC_019489(34) | 40.7 |
| | 83.9 | 150 | 87 | 97.7 | 2089314-2173290 | Gordon_Kita_NC_031233(12) | 41.4 |
| CMPG5300 | 41.9 | 150 | 54 | 100 | 999151-1041084 | Lactob_Sha1_NC_019489(25) | 40.4 |
| | 48.3 | 100 | 61 | 91.8 | 2118840-2167213 | Lactob_phig1e_NC_004305(9) | 41.2 |
| | 34.7 | 110 | 46 | 97.8 | 3239310-3274049 | Strept_phiARI0746_NC_031907(11) | 41.9 |
| DF | 39.6 | 150 | 49 | 95.9 | 460-40154 | Lactob_Sha1_NC_019489(27) | 42.0 |
| | 54.7 | 150 | 61 | 100 | 459006-513755 | Lactob_phig1e_NC_004305(27) | 42.6 |
| | 16.5 | 90 | 22 | 100 | 1022096-1038662 | Lactob_Sha1_NC_019489(3) | 41.1 |
| | 140.5 | 150 | 185 | 96.2 | 2125401-2265944 | Lactoc_PLgT_1_NC_031016(22) | 41.5 |
| | 41.5 | 150 | 51 | 100 | 3166433-3207995 | Lactob_Sha1_NC_019489(25) | 41.7 |
| | 27.5 | 130 | 30 | 100 | 3396222-3423810 | Lactob_Sha1_NC_019489(27) | 41.7 |

| | | | | | | | |
|---|---|---|---|---|---|---|---|
| dm | 44.2 | 100 | 62 | 100 | 2008496-2052753 | Strept_9871_NC_031069(12) | 42.3 |
| | 49.7 | 150 | 62 | 100 | 2130341-2180071 | Lactob_Sha1_NC_019489(34) | 41.8 |
| | 50.1 | 100 | 51 | 100 | 3253989-3304157 | Oenoco_phiS13_NC_023560(15) | 42.0 |
| DOMLa | 16.9 | 80 | 23 | 91.3 | 37804-54802 | Lactob_Sha1_NC_019489(3) | 42.4 |
| | 38.9 | 130 | 55 | 96.3 | 513739-552681 | Bacill_vB_BhaS_171_NC_030904(14) | 41.5 |
| | 42.7 | 150 | 52 | 98.0 | 1021684-1064390 | Lactob_Sha1_NC_019489(18) | 41.5 |
| GB-LP1 | 43 | 80 | 26 | 92.3 | 2040278-2083314 | Staphy_StB20_like_NC_028821(3) | 43.5 |
| HFC8 | 51.8 | 130 | 68 | 98.5 | 842692-894565 | Lactob_PLE2_NC_031036(11) | 40.8 |
| | 17.5 | 70 | 20 | 90.0 | 889402-906990 | Lactob_Sha1_NC_019489(3) | 41.6 |
| | 41.2 | 70 | 35 | 57.1 | 2374916-2416127 | Entero_phiEF24C_NC_009904(3) | 45.0 |
| | 48.4 | 150 | 56 | 100 | 2950226-2998645 | Lactob_Sha1_NC_019489(23) | 41.3 |
| *pMK01* | 9.3 | 50 | 12 | 100 | 55975-65313 | Entero_phi92_NC_023693(4) | 39.8 |
| *pMK03* | 32.4 | 100 | 18 | 83.3 | 3489-35957 | Staphy_SPbeta_like_NC_029119(2) | 42.1 |
| *pMK04* | 17.8 | 100 | 24 | 100 | 27647-45467 | Bacill_SalinJah_NC_031034(1) | 41.7 |
| *pMK07* | 11.2 | 40 | 17 | 94.1 | 1-11265 | Lactob_Sha1_NC_019489(8) | 38.8 |
| JBE245 | 16 | 80 | 22 | 95.4 | 36006-52093 | Entero_Ec_ZZ2_NC_031260(3) | 41.4 |
| | 67 | 120 | 72 | 88.8 | 570558-637646 | Gordon_Cozz_NC_030941(12) | 42.3 |
| | 42.6 | 150 | 55 | 100 | 1254805-1297448 | Lactob_Sha1_NC_019489(28) | 40.4 |
| | 41.7 | 90 | 24 | 95.8 | 2171278-2213069 | Staphy_StB20_like_NC_028821(3) | 43.1 |
| JBE490 | 14.4 | 60 | 19 | 94.7 | 37371-51778 | Staphy_IME_SA4_NC_029025(2) | 41.6 |
| | 28 | 90 | 25 | 60.0 | 1015717-1043748 | Entero_phi92_NC_023693(4) | 39.1 |
| | 45.8 | 150 | 56 | 100 | 1176972-1222828 | Lactob_Sha1_NC_019489(30) | 41.1 |
| | 68.2 | 150 | 76 | 89.4 | 2120757-2189051 | Clostr_phiCT453A_NC_028991(11) | 41.1 |
| JDM1 | 22.6 | 100 | 28 | 89.2 | 38452-61144 | Entero_EFRM31 | 42.6 |
| | 38.9 | 120 | 52 | 98.0 | 513507-552448 | Lister_B025 | 41.5 |
| | 42.7 | 140 | 51 | 98.0 | 1021445-1064151 | Lactob_Sha | 41.5 |
| | 8.9 | 30 | 8 | 87.5 | 1074721-1083640 | Megavi_lba | 41.4 |
| | 16.1 | 20 | 7 | 85.7 | 1094265-1110371 | Escher_phAPEC8 | 41.2 |

| | | | | | | | |
|---|---|---|---|---|---|---|---|
| KLDS1.0391 | 83.6 | 150 | 86 | 91.8 | 477775-561427 | Clostr_phiCT453A_NC_028991(11) | 41.4 |
| | 43.5 | 150 | 55 | 98.1 | 2473939-2517476 | Lactob_Sha1_NC_019489(21) | 41.7 |
| KP | 54.7 | 150 | 61 | 100 | 83886-138635 | Lactob_phig1e_NC_004305(27) | 42.6 |
| | 16.5 | 90 | 22 | 100 | 646975-663541 | Lactob_Sha1_NC_019489(3) | 41.1 |
| | 140.5 | 150 | 185 | 96.2 | 1750262-1890805 | Lactoc_PLgT_1_NC_031016(22) | 41.5 |
| | 41.5 | 150 | 51 | 100 | 2791295-2832857 | Lactob_Sha1_NC_019489(25) | 41.7 |
| | 44.7 | 150 | 55 | 96.3 | 3021084-3065860 | Lactob_Sha1_NC_019489(35) | 41.6 |
| LP2 | 63.1 | 150 | 60 | 100 | 1801216-1864354 | Lactob_Sha1_NC_019489(26) | 40.9 |
| | 28.5 | 20 | 27 | 100 | 1839018-1867612 | Lactob_Sha1_NC_019489(11) | 40.5 |
| | 2.2 | 130 | 63 | 100 | 2162379-2214628 | Lactob_Lj965_NC_005355(16) | 41.4 |
| LP3 | 40.7 | 150 | 50 | 100 | 1841900-1882608 | Lactob_Sha1_NC_019489(18) | 40.5 |
| | 52.8 | 100 | 51 | 88.2 | 2161070-2213963 | Oenoco_phi9805_NC_023559(16) | 41.5 |
| *pLB301* | 10.7 | 30 | 11 | 81.8 | 20666-31374 | Paenib_Tripp_NC_028930(3) | 40.6 |
| | 7.7 | 50 | 9 | 66.6 | 37886-45602 | Bacill_G_NC_023719(2) | 40.9 |
| *pLB302* | 3.9 | 10 | 7 | 100 | 13579-17500 | Bacill_SP_15_NC_031245(1) | 35.8 |
| LPL-1 | 45.5 | 150 | 52 | 96.1 | 955654-1001243 | Lactob_phig1e_NC_004305(16) | 41.0 |
| | 47 | 150 | 55 | 96.3 | 2042663-2089739 | Lactob_Sha1_NC_019489(30) | 41.0 |
| | 38.2 | 100 | 50 | 100 | 2572537-2610749 | Lactob_PLE2_NC_031036(11) | 41.4 |
| LY-78 | 39.6 | 150 | 49 | 100 | 159428-199073 | Lactob_Sha1_NC_019489(19) | 40.7 |
| | 20.3 | 60 | 24 | 100 | 2957873-2978264 | Staphy_StB20_like_NC_028821(3) | 41.9 |
| LZ206 | 46 | 120 | 55 | 100 | 329210-375273 | Lactob_phig1e_NC_004305(11) | 41.7 |
| | 41.4 | 150 | 54 | 100 | 654343-695793 | Lactob_Sha1_NC_019489(35) | 40.9 |
| | 21.5 | 50 | 9 | 100 | 1452463-1474036 | Prochl_P_SSM2_NC_006883(2) | 39.4 |
| | 61.7 | 100 | 52 | 100 | 1965209-2026992 | Strept_phiARI0746_NC_031907(12) | 42.9 |
| LZ227 | 74.2 | 140 | 79 | 93.6 | 289629-363863 | Staphy_CNPx_NC_031241(12) | 41.9 |
| | 44.4 | 120 | 63 | 98.4 | 629219-673685 | Lactob_Sha1_NC_019489(13) | 41.4 |
| | 16 | 60 | 24 | 95.8 | 2390301-2406374 | Strept_315.2_NC_004585(3) | 41.6 |
| *LZ227p2* | 17.9 | 50 | 16 | 68.7 | 949-18850 | Staphy_SPbeta_like_NC_029119(2) | 43.5 |

| | | | | | | | |
|---|---|---|---|---|---|---|---|
| *LZ227p4* | 11.1 | 80 | 20 | 100 | 2498-13600 | Entero_EF62phi_NC_017732(2) | 39.5 |
| | 8.2 | 50 | 9 | 100 | 26768-35052 | Bacill_G_NC_023719(2) | 41.9 |
| | 10.3 | 60 | 16 | 100 | 68782-79088 | Entero_EF62phi_NC_017732(2) | 38.0 |
| LZ95 | 39.8 | 120 | 55 | 100 | 1200693-1240586 | Lactob_phijl1_NC_006936(18) | 41.3 |
| | 54.9 | 120 | 68 | 95.5 | 1253004-1307933 | Oenoco_phiS13_NC_023560(16) | 41.3 |
| | 22.1 | 50 | 12 | 83.3 | 2597662-2619810 | Strept_phiARI0131_2_NC_031941(3) | 40.6 |
| *LZ95p1* | 7.7 | 50 | 9 | 66.6 | 1553-9310 | Bacill_G_NC_023719(2) | 40.9 |
| | 10.7 | 30 | 11 | 81.8 | 16676-27382 | Entero_phiEF24C_NC_009904(2) | 40.4 |
| MF1298 | 42.3 | 80 | 34 | 55.8 | 564136-606492 | Entero_phiEF24C_NC_009904(3) | 44.9 |
| | 43.7 | 150 | 54 | 100 | 1153027-1196816 | Lactob_Sha1_NC_019489(35) | 40.5 |
| | 53.1 | 140 | 62 | 96.7 | 2089026-2142166 | Oenoco_phiS13_NC_023560(16) | 41.8 |
| *pMF1298-2* | 7.7 | 50 | 9 | 66.6 | 1553-9305 | Bacill_G_NC_023719(2) | 40.9 |
| | 7.9 | 20 | 8 | 87.5 | 18623-26523 | Entero_phiEF24C_NC_009904(2) | 40.7 |
| *unnamed4* | 18.7 | 40 | 10 | 80.0 | 3733-22515 | Erysip_SE_1_NC_029078(2) | 42.8 |
| NCU116 | 42.4 | 120 | 50 | 98.0 | 2243626-2286096 | Lactoc_98201_NC_031064(12) | 41.6 |
| | 12.4 | 30 | 12 | 100 | 2281958-2294400 | Lactob_Ld25A_NC_025415(3) | 43.1 |
| | 44.4 | 150 | 55 | 100 | 2577765-2622249 | Lactob_Sha1_NC_019489(39) | 40.1 |
| | 40.5 | 110 | 55 | 90.9 | 3214066-3254657 | Lactob_PLE2_NC_031036(12) | 40.5 |
| | 10.7 | 50 | 11 | 81.8 | 3288056-3298762 | Entero_phiEF24C_NC_009904(2) | 40.4 |
| PC520 | 45.2 | 110 | 63 | 98.4 | 914787-960005 | Strept_phiARI0131_1_NC_031901(11) | 41.2 |
| | 87.2 | 150 | 104 | 100 | 1605018-1692270 | Lactob_Sha1_NC_019489(35) | 40.8 |
| | 52.2 | 130 | 63 | 98.4 | 2578764-2631005 | Lactob_Lj965_NC_005355(16) | 41.4 |
| *p1* | 56.2 | 130 | 71 | 63.3 | 1-56283 | Bacill_G_NC_023719(3) | 41.0 |
| *p2* | 51.8 | 100 | 50 | 62.0 | 1-51862 | Entero_phiEF24C_NC_009904(3) | 38.6 |
| RI-113 | 26 | 50 | 14 | 92.8 | 869253-895346 | Oenoco_phiS13_NC_023560(1) | 42.2 |
| | 42.4 | 150 | 50 | 98.0 | 1640596-1683017 | Lactob_Sha1_NC_019489(22) | 41.7 |
| | 58.1 | 140 | 61 | 100 | 2118661-2176793 | Lactob_phig1e_NC_004305(12) | 42.0 |
| | 42.5 | 110 | 55 | 96.3 | 2199369-2241939 | Lactob_PLE2_NC_031036(14) | 41.7 |

| | | | | | | | |
|---|---|---|---|---|---|---|---|
| *pRI113_1* | 7.4 | 50 | 11 | 100 | 17567-25016 | Lactob_phiAT3_NC_005893(2) | 39.7 |
| *pRI113_2* | 34.8 | 150 | 40 | 100 | 5359-40208 | Bacill_G_NC_023719(4) | 43.0 |
| *pRI113_6* | 9.5 | 50 | 10 | 100 | 134-19659 | Staphy_SPbeta_like_NC_029119(2) | 39.8 |
| SRCM102022 | 43.1 | 90 | 57 | 92.9 | 562955-606137 | Weisse_WCP30_NC_031101(12) | 40.0 |
| | 19.7 | 30 | 6 | 100 | 1042949-1062655 | Entero_phi92_NC_023693(4) | 37.3 |
| | 56.1 | 150 | 53 | 100 | 1801989-1858119 | Lactob_Sha1_NC_019489(38) | 40.7 |
| | 50.5 | 130 | 63 | 96.8 | 2145103-2195636 | Lactob_PLE2_NC_031036(12) | 41.9 |
| *pPL2022-1* | 11.5 | 40 | 8 | 62.5 | 24963-36493 | Entero_N15_NC_001901(2) | 45.4 |
| | 21.6 | 50 | 13 | 84.6 | 43140-64799 | Clostr_phiCTC2A_NC_030949(3) | 38.9 |
| TMW 1.25 | 34.3 | 90 | 35 | 100 | 2089466-2123767 | Oenoco_phiS13_NC_023560(16) | 42.2 |
| | 32.9 | 80 | 26 | 96.1 | 2132644-2165569 | Lactob_Sha1_NC_019489(3) | 40.8 |
| *pL125-2* | 38.5 | 90 | 45 | 68.8 | 1-38549 | Bacill_G_NC_023719(3) | 40.6 |
| TMW 1.277 | 34.3 | 90 | 35 | 100 | 2044017-2078318 | Oenoco_phiS13_NC_023560(16) | 42.2 |
| | 32.9 | 80 | 26 | 100 | 2087195-2120120 | Lactob_Sha1_NC_019489(3) | 40.8 |
| *pL1277-1* | 64.5 | 150 | 68 | 58.8 | 1-64533 | Staphy_SPbeta_like_NC_029119(2) | 39.5 |
| *pL1277-4* | 30.6 | 100 | 35 | 71.4 | 1-30691 | Lactob_phiAT3_NC_005893(2) | 36.9 |
| *pL1277-7* | 10.3 | 20 | 8 | 75.0 | 28429-38743 | Bacill_G_NC_023719(2) | 42.8 |
| TMW 1.708 | 38.9 | 110 | 52 | 96.1 | 505640-544542 | Lactob_PLE2_NC_031036(13) | 41.5 |
| | 57.1 | 150 | 53 | 100 | 1774602-1831742 | Lactob_Sha1_NC_019489(40) | 40.7 |
| | 42.3 | 80 | 26 | 96.1 | 2098921-2141275 | Lactob_Sha1_NC_019489(3) | 43.5 |
| *pL1708-1* | 17.1 | 50 | 13 | 100 | 899-18080 | Shigel_Sf6_NC_005344(1) | 36.6 |
| *pL1708-2* | 45.6 | 120 | 55 | 56.3 | 1-45679 | Clostr_phiCD6356_NC_015262(2) | 39.8 |
| *pL1708-3* | 6.2 | 20 | 9 | 66.6 | 5956-12173 | Pseudo_NP1_NC_031058(5) | 38.1 |
| TMW 1.1623 | 44.2 | 150 | 50 | 100 | 1129520-1173731 | Lactob_Sha1_NC_019489(35) | 41.5 |
| | 47.3 | 100 | 51 | 92.1 | 2074572-2121906 | Oenoco_phiS13_NC_023560(16) | 41.2 |
| *pL11623-1* | 6.8 | 40 | 9 | 100 | 12184-19066 | Shigel_SfII_NC_021857(2) | 36.5 |
| | 7.1 | 50 | 12 | 100 | 34360-41556 | Entero_fiAA91_ss_NC_022750(2) | 39.6 |
| *pL11623-3* | 13 | 70 | 15 | 100 | 13332-26423 | Paenib_Tripp_NC_028930(3) | 40.8 |

| | | | | | | | |
|---|---|---|---|---|---|---|---|
| | 7.7 | 40 | 9 | 100 | 31404-39161 | Bacill_G_NC_023719(2) | 41.0 |
| *pL11623-4* | 7.1 | 20 | 7 | 85.7 | 33278-40454 | Entero_phiEF24C_NC_009904(2) | 41.9 |
| WCFS1 | 43.8 | 150 | 63 | 63.4 | 589962-633804 | Lactob_phig1e_NC_004305(11) | 40.8 |
| | 69.3 | 150 | 82 | 65.8 | 2156164-2225482 | Lactob_phig1e_NC_004305(11) | 41.0 |
| Zhang-LL | 41.1 | 70 | 34 | 55.8 | 520368-561480 | Entero_phiEF24C_NC_009904(3) | 45.0 |
| | 66.8 | 150 | 84 | 97.6 | 1976544-2043398 | Lactob_phig1e_NC_004305(21) | 41.0 |
| ZJ316 | 17.5 | 20 | 9 | 66.6 | 259978-1277515 | Ostreo_2 | 39.0 |
| | 39.8 | 110 | 52 | 88.4 | 1434410-1474285 | Lister_B025 | 40.9 |
| | 41.4 | 150 | 53 | 92.4 | 2079541-2121002 | Lactob_Sha1 | 40.9 |
| | 47.4 | 150 | 59 | 81.3 | 2398693-2446136 | Lactob_phig1e | 41.7 |
| | 9.4 | 40 | 9 | 88.8 | 2891252-2900663 | PROBrucel_1330 | 46.4 |
| ZS2058 | 47.6 | 130 | 55 | 100 | 1340641-1388292 | Lactob_PLE2_NC_031036(10) | 41.3 |
| BLS41 | 40.5 | 150 | 50 | 100 | 631838-672404 | Lactob_Sha1_NC_019489(18) | 40.6 |
| | 47.1 | 100 | 54 | 100 | 958026-1005184 | Oenoco_phi9805_NC_023559(17) | 41.2 |
| | 39.5 | 120 | 58 | 100 | 3207743-3247302 | Bacill_vB_BhaS_171_NC_030904(14) | 41.4 |
| *pLPBLS41_1* | 38.9 | 110 | 50 | 96.0 | 57-38975 | Clostr_phiCT19406B_NC_030947(13) | 41.4 |
| *pLPBLS41_2* | 12.4 | 70 | 15 | 100 | 14162-26571 | Paenib_Tripp_NC_028930(3) | 40.5 |
| | 10.7 | 70 | 12 | 100 | 30091-40840 | Paenib_Tripp_NC_028930(3) | 40.5 |
| *pLPBLS41_3* | 25.7 | 50 | 11 | 100 | 16045-41769 | Plankt_PaV_LD_NC_016564(1) | 41.3 |
| CGMCC 1.557 | 58 | 150 | 53 | 98.1 | 1283184-1341257 | Lactob_Sha1_NC_019489(20) | 40.6 |
| | 47.9 | 150 | 56 | 100 | 1626971-1674892 | Lactob_phig1e_NC_004305(25) | 41.9 |
| | 28.4 | 60 | 21 | 100 | 1352718-1381129 | Staphy_phiPV83_NC_002486(3) | 40.5 |
| *pLp1* | 22.8 | 70 | 15 | 86.6 | 41562-64457 | Entero_N15_NC_001901(2) | 39.4 |
| P-8 | 48.9 | 150 | 52 | 100 | 1687548-1736475 | Lactob_Sha1_NC_019489(27) | 41.0 |
| | 42 | 90 | 55 | 96.3 | 2022535-2064626 | Weisse_WCP30_NC_031101(8) | 40.6 |
| | 28.6 | 80 | 22 | 95.4 | 2063771-2092419 | Lactob_Sha1_NC_019489(4) | 41.0 |
| *LBPp2* | 6.8 | 40 | 8 | 75.0 | 1598-28449 | Clostr_phiCD119_NC_007917(1) | 38.7 |
| *LBPp3* | 7.5 | 50 | 11 | 63.6 | 5741-13254 | Natria_PhiCh1_NC_004084(1) | 41.0 |

| | | | | | | | |
|---|---|---|---|---|---|---|---|
| SRCM100434 | 40.9 | 150 | 50 | 100 | 1323097-1364034 | Lactob_Sha1_NC_019489(25) | 40.3 |
| ST-III | 39.9 | 150 | 48 | 100 | 1799567-1839527 | Lactob_Sha1 | 40.8 |
| | 50.5 | 120 | 58 | 81.0 | 2139385-2189972 | Lactob_Lj965 | 41.3 |
| *pST-III* | 8.5 | 40 | 7 | 85.7 | 1553-10140 | Bacill_G_NC_023719(2) | 40.8 |
| | 18.5 | 60 | 11 | 81.8 | 10140-28710 | Entero_phiEF24C_NC_009904(2) | 39.8 |
| TS12 | 36.3 | 120 | 41 | 100 | 1042-37425 | Lactob_PLE3_NC_031125(7) | 42.0 |
| | 41.8 | 150 | 55 | 100 | 324054-365949 | Lactob_Sha1_NC_019489(24) | 40.5 |
| | 44.8 | 90 | 65 | 100 | 2589973-2634846 | Lactob_phig1e_NC_004305(14) | 41.5 |
| *pLP12-2* | 11.5 | 100 | 23 | 100 | 6565-18108 | Lactob_phiAT3_NC_005893(2) | 38.5 |
| X7021 | 30.7 | 50 | 17 | 70.5 | 791071-821829 | Lactob_jlb1_NC_024206(2) | 40.6 |
| | 56.3 | 150 | 57 | 96.4 | 1745969-1802357 | Lactob_Sha1_NC_019489 (29) | 41.1 |
| | | 150 | 79 | 92.4 | 2069489-2150023 | Lactob_phig1e_NC_004305(22) | 42.6 |
