# Supplementary material for: Comparative Genomic Analysis of Lactobacillus plantarum: An Overview
Source: Int J Genomics. 2019 Apr 10;2019:4973214. doi: 10.1155/2019/4973214 (PMC6481158; doi:10.1155/2019/4973214)
Supplement: Supplementary 3 — CRISPR sequences present in the analyzed genomes. [file 4973214.f3.pdf]

Table S3. CRISPR sequences obtained by CrisprFinder.

Strain	Start–end	Length (bp)	Consensus Sequence	Spacer sequences
CLP0611	114992-115687	695	**GTCTTGAATAGTAGTCATATCAAACAGGTTTAGAAC**	CAACAGACTCCTGCTGGGGTTGCCTCAGTT
				TCGAATTAATGCTGACGCCCAGTTATTGAT
				AAACGTCTTTAGCGTAGTCATTCATTGGCT
				AAACACCGTTAACGGTCTTACCGTGTCGAT
				ACGTCGGCGAAATCTTCACCACGCCGATTA
				TTGATGACTCTTCTGGGGCACCAACAAGTT
				TATTGAGTGCAGCGTTGTTTGCGAGCGTCC
				GTACAGTGTCGCTCAACGTCTATCACTCCC
				GGTATGGAGTACAACAAGGAACAAGACACG
				GATGCTGAGGAAACGAAGCATAGAACGTTT
LY-78	1823736-1824036	300	**GTTCTAAACCTGTTTGATATGACTACTATTCAAGAC**	AAACGTTCTATGCTTCGTTTCCTCAGCATC
				TAATCGGCGTGGTGAAGATTTCGCCGACGT
				AGCCAATGAATGACTACGCTAAAGACGTTT
				TACGGTACTCAATTACGATTAGATAATACAA
LZ206	2416755-2417252	497	**GTTCTAAACCTGTTTGATATGACTACTATTCAAGAC**	AAACGTTCTATGCTTCGTTTCCTCAGCATC
				CGTAAATAGGTTGTAAACGTTCAGGTCTGA
				TAATACGATCCATTGACTCCGCTAGTGACA
				TATTCTCGTTTATCGCTTTTAGACGTGTTA
				GTTTGGACTGGTTTATCAAGCAACAAGTTG
				AAGCGCGGGATCACGTGCAACAGCACCATC
				ACGTCGGCAAACTGTTCCAATCCTTGACAT
LZ227	2311451-2312014	563	**GTTCTAAACCTGTTTGATATGACTACTATTCAAGAC**	AAACGTTCTATGCTTCGTTTCCTCAGCATC
				GGCATGTTAGTAGTTTAGGCGCCCTCCAAA

				TGCTTTCGTTTTTAAGTTGTCCGCCTAACT<br>TCGCGCCGCGAAACATTGGACAGACTTAGT<br>TGATCAAACAGTTTATAACAAGTTAAAAGA<br>AGAAGATATATGTTCCAGATACTAATTCTG<br>TGGTTACGACGCGTGGGGGCTAACGCCTAC<br>GATGATGAGCGTATGATTCAGCAAGCCATG
MF1298	116735-118846	2111	**GTCTTGAATAGTAGTCATATCAAACAGGTTTAGAAC**	AAAATGGATTTCTGAGCATTACTGTCCGAC<br>GACTATACCAATGAGGTCGAAGCATGGTTA<br>CACAAGGCCACCGAGGAAGCCGGCGAGCTA<br>TAGGTTTACTCATGGTAAATCCTCCTATGT<br>AAAATAAATTTAAGGTTGCGCAACACAATG<br>AACTCATCATAAATGACGTCTTTTACCGAG<br>TTAGGTTGAGCTGGATCGGGATCAGGATCG<br>TCTGTTGTTTAATTTGTTTTAGATTGTTAC<br>ACTAAAGCCGGGGTATACAGCCCCGAAGAA<br>AAAATAGTTCGACGAAAAAGCCGAAAGAGA<br>GCGGCCACGACCGCCATGGGTGTCAGCGCC<br>TCGGATAACTTAGCGGCGGCGTATCCCTGC<br>ATAGTGACAGCATCTGTTTTCGGACCAATC<br>GAGGCTTGCACTAGTGAGTTCAATCGTTAT<br>TGTCCATACGAGCAGCGTTGGCACTTATTC<br>GTATTATTATCAACGTCCCGCATTGCGTTA<br>AGGATATATGAAATTAGTACATGTACTAGT<br>ACGTCTTTCAGCCCAGTAAACTGCTCAAGT<br>TTGAATACCATTCCTTGTTTATACTCCATC<br>AGTTCATAATCATATGATCTAAGTGACGGT<br>GGCCGGGTTAATCTTAATGTTTTATGTTCT<br>CAATCAGAAAGAAGATGACGACTATAATGC

				GACTTATACCAGCAGTACCGAAGACGGTTA AACTGGGAGCGGTCAACACCCCAGGCTGTG ACGCAAAGCCAGCCCTAGTCATGAGGTCAT GTTGCAACTTTATCCTTGTCACTTTCAACA CAATACCATAGTAGTCAATTATTACACGTCG TCTTGAATAGTAGTCAATTATTACACGTC GCCAAACAACATTGCAGAGAGCTAGTGCTT GTGACGTGCTCCCATGTGACCCGAATTGAC CGACTCTAATGGATATTTTCAATCATAGCA GATGGTGCTGTTGCACGTGATCCCGCGCTT
ZJ316	359930-360361	431	**GTCTTGAATAGTAGTCATATCAAACAGGTTTAGAAC**	ATGTCAAGGATTGGAACAGTTTGCCGACGT CTTGAAGAAGTTAAGGCACAAACTCCAACG TAACACGTCTAAAAGCGATAAACGAGAATA TGTCACTAGCGGAGTCAATGGATCGTATTA TCAGACCTGAACGTTTACAACCTATTTACG GATGCTGAGGAAACGAAGCATAGAACGTTT
ZS2058	2563734–2564693	959	**GTCTTGAATAGTAGTCATATCAAACAGGTTTAGAAC**	GACGAAATTACAAAAGACCGTCATAAGCGT CGGCAAGTAATCAGCTAGGAGGTTCGCGCC TATGGAATGGGTCAAGTTTTTCACTCAATC AATGATTATGTGGACGAACATAATCAGATG ACGCTATGCGTTCGTATGTAGCAACTCATA TGTCGCGCTGCGCATTAGCCGTAATCAACT CCACCAGTTATCGCGATTACTTAACTTATT GCCTAGGTCAAGAACTAACTAAAGAGTTAG TGTCACTTTCTAATGAACTTGCAACCATCA CCGGTCGCCACCGGGACAAGCCCACAAAGA

				ACATTGATGACTATTTCGGCAAAGAATATT<br>AACACTAGTAAATTGCCACATGGCAATGTC<br>AATGCCAGTCCTTTGTATTCACTTTTGTTA<br>GATGCTGAGGAAACGAAGCATAGAACGTTT
CGMCC 1.557	2774673-2775303	630	**GTCTTGAATAGTAGTCATATCAAACAGGTTTAGAAC**	TTGAAGTCCCAGCCGTGAACCAGTTAATGA<br>TCAAATTAAGAATGCCGTAGCCACAATTAC<br>GGACGCATCGGGGTATATTGCTGACGAAGA<br>AGAACGGTACGTATGAGTTAACACCTAAAT<br>TTGTATTATCTAATCGTAATTGAGTACCGTA<br>GCTATACTATAAACATATATAAGGAAAGGA<br>GATTAAATGCAAGTACTGTCTTTCGAATAT<br>ACGTCGGCGAAATCTTCACCACGCCGATTA<br>GATGCTGAGGAAACGAAGCATAGAACGTTT
TS12	2154877-2155571	694	**GTCTTGAATAGTAGTCATATCAAACAGGTTTAGAAC**	GATTCTAGTTCGCTTTGCTGAGCCTTAAGA<br>CACGGACGCTCTACACATTCATGCCGTCAT<br>CTAACCAAATTTATCTGTTTGGGAATGGAG<br>CTTCTAGTCCATGTTTTTGTACGATTTCAT<br>TAAGACCCCCAAGATACCGGCGGCCGCTTG<br>TTAAGTGTTTTAGCACAACTCCAATTTATT<br>TAAACCGCAGCAGTAACGTATGCACGAAC<br>TACAACAATAGCAGCCGTGCCACAAGTTGT<br>AGTTTTCTTTACCAACGAACCCAATACCAC<br>GCGTTGACGAGCTTTAATCTCTCACGCAAC
