# Supplementary material for: Comparative Genomic Analysis of Lactobacillus plantarum: An Overview
Source: Int J Genomics. 2019 Apr 10;2019:4973214. doi: 10.1155/2019/4973214 (PMC6481158; doi:10.1155/2019/4973214)
Supplement: Supplementary 4 — Detailed information on the pan-genome analysis. [file 4973214.f4.pdf]

Table S4. Orthologous genes shared between *L. plantarum* WCFS1 and other thirty-nine strains on region 2 of the lifestyle island. Strains showed less than 10 OGs were not represented.

**Organism name**	**OGs**	**G+C %**
L. plantarum 10CH	31	43.4
L. plantarum 5-2	56	41.7
L. plantarum ATCC 8014	65	41.8
L. plantarum B21	56	41.8
L. plantarum BDGP2	38	41.3
L. plantarum C410L1	17	44.9
L. plantarum CAUH2	39	41.3
L. plantarum CLP0611	31	40.8
L. plantarum DF	39	41.3
L. plantarum dm	39	41.3
L. plantarum DOMLa	65	41.9
L. plantarum GB-LP1	24	44.1
L. plantarum HFC8	20	44.8
L. plantarum JBE245	56	41.7
L. plantarum JBE490	14	43.8
L. plantarum JDM1	65	41.9
L. plantarum KLDS1.0391	20	44.2
L. plantarum KP	39	41.3
L. plantarum LM1004	28	44.2
L. plantarum LP3	42	40.2
L. plantarum LPL-1	34	40.2
L. plantarum LY-78	30	41.1
L. plantarum LZ206	24	44.1
L. plantarum LZ227	24	44.1
L. plantarum MF1298	31	43.4
L. plantarum NCU116	59	41.6
L. plantarum PC520	39	41.3
L. plantarum SRCM102022	60	41.6
L. plantarum TMW 1.25	25	44.3
L. plantarum TMW 1.277	25	44.3
L. plantarum TMW 1.708	28	42.9
L. plantarum TMW 1.1623	55	41.6
L. plantarum WCFS1	179	41.9
L. plantarum X7021	28	40.2
L. plantarum Zhang-LL	18	43.4
L. plantarum ZJ316	24	44.1
L. plantarum ZS2058	37	40.5
L. plantarum subsp. *plantarum* CGMCC 1.557	31	40.8
L. plantarum subsp. *plantarum* P-8	24	44.4

L. plantarum subsp. *plantarum* TS12	38	41.4
